# The Role of Serotonin in the Regulation of Patience and Impulsivity

**DOI:** 10.1007/s12035-012-8232-6

**Published:** 2012-01-20

**Authors:** Katsuhiko Miyazaki, Kayoko W. Miyazaki, Kenji Doya

**Affiliations:** 1 Neural Computation Unit, Okinawa Institute of Science and Technology, Okinawa, 904-0412 Japan; 2 Computational Neuroscience Laboratories, Advanced Telecommunications Research Institute International, Kyoto, 619-0288 Japan

**Keywords:** Serotonin, Delayed reward, Impulsivity, Patience, Dorsal raphe nucleus, Waiting to obtain reward

## Abstract

Classic theories suggest that central serotonergic neurons are involved in the behavioral inhibition that is associated with the prediction of negative rewards or punishment. Failed behavioral inhibition can cause impulsive behaviors. However, the behavioral inhibition that results from predicting punishment is not sufficient to explain some forms of impulsive behavior. In this article, we propose that the forebrain serotonergic system is involved in “waiting to avoid punishment” for future punishments and “waiting to obtain reward” for future rewards. Recently, we have found that serotonergic neurons increase their tonic firing rate when rats await food and water rewards and conditioned reinforcer tones. The rate of tonic firing during the delay period was significantly higher when rats were waiting for rewards than for tones, and rats were unable to wait as long for tones as for rewards. These results suggest that increased serotonergic neuronal firing facilitates waiting behavior when there is the prospect of a forthcoming reward and that serotonergic activation contributes to the patience that allows rats to wait longer. We propose a working hypothesis to explain how the serotonergic system regulates patience while waiting for future rewards.

## Introduction

Serotonin (5-hydroxytryptamine, 5-HT) has been implicated in a variety of motor, cognitive, and affective functions [1–3], such as locomotion, sleep–wake cycles, and mood disorders. A large number of studies have shown that reduced levels of 5-HT in the central nervous system promote impulsive behaviors [4–8], including impulsive action (i.e., the failure to suppress inappropriate actions) and impulsive choice (i.e., the choice of small, immediate rewards over larger, delayed rewards). However, recent studies of the effects of manipulating 5-HT levels on impulsivity have reported mixed results [9–27].

The aim of this article is to propose a new concept of the role of 5-HT system in waiting for delayed rewards, based on our recent microdialysis and unit recording studies [28, 29]. This article is organized as follows. First, we present an overview of the types of impulsive behaviors that are involved in the 5-HT system. The depletion of forebrain serotonin transmission induces impulsive action as assessed by the five-choice serial reaction time task (5-CSRTT), which is commonly used to measure impulsive action [13–16, 19–21, 25]. Modulating central 5-HT transmission also influences impulsive choice as assessed using the commonly used delay-discounting task. However, contradictory results have been reported [9–12, 17, 18, 22–24, 26, 27].

Second, we review recent microdialysis and unit recording studies that examined the 5-HT neural activity of behaving animals. We found previously that 5-HT efflux in the dorsal raphe nucleus (DRN), the primary origin of 5-HT projections to the forebrain [1], increases when rats perform a task that requires waiting for a delayed reward [28]. We also found that 5-HT neurons in the DRN exhibit an increase in their tonic firing rate when rats await delayed rewards and that these neurons cease firing before rats stop waiting for a reward that has been delayed for too long [29]. These results demonstrate an association between dorsal raphe 5-HT activation and the waiting behavior that is associated with a delayed reward.

Third, we propose the new concept “waiting to obtain reward”, which means that animals reduce their behavioral activity to obtain a forthcoming reward. We hypothesize that an increase in 5-HT neural activity during waiting for a delayed reward contributes to the regulation of the “waiting to obtain reward”. Classic theories suggest that central 5-HT neurons are involved in the behavioral inhibition that is associated with the prediction of negative rewards or punishment [30–33]. We propose that the 5-HT system is involved both in “waiting to avoid punishment” for future punishment and in “waiting to obtain reward” for future reward. Some forms of impulsive action that have been studied pharmacologically or by lesion studies can be explained by the concept of “waiting to obtain reward”.

Finally, we propose a neural mechanism of patience that is related to the “waiting to obtain reward”. Interactions among the orbitofrontal cortex (OFC), medial prefrontal cortex (mPFC), nucleus accumbens (NAcc), and 5-HT neurons in the DRN are closely related to the “waiting to obtain a reward”.

## Serotonin and Impulsivity

**Fig. 1:**
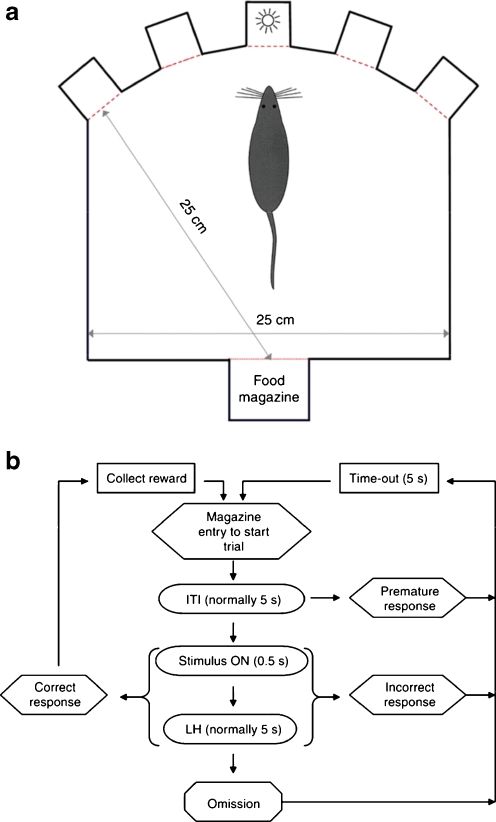
Apparatus and behavioral procedure of the five-choice serial reaction time task. **a** Schematic diagram of the five-choice serial reaction time task chamber, showing the spatial arrangement of the five response apertures in relation to the food magazine. **b** Possible trial sequences of the five-choice serial reaction time task. A trial is initiated when the rat enters the food magazine. A brief light stimulus is then presented in one of five possible apertures after a 5-s inter-trial interval (ITI). The rat is required to scan the five apertures for the appearance of the light stimulus and then respond in the “correct” aperture with a nose-poke response to earn a single food pellet. If the rat responds before the stimulus (“premature response”) or in an adjacent incorrect aperture (“incorrect response”), a 5-s time-out (TO) period is introduced in which the house light is extinguished and no food reward is provided. A failure to respond within the limited hold (LH) period results in an “omission” and subsequent 5-s TO period. After collecting the reward or at the end of the TO period, a head entry in the food magazine initiates a new trial. Modified from [34]

An altered functionality of the serotonergic system has been implicated in impulsivity. Impulsivity can be divided broadly into impulsive action and impulsive choice [5] (Table 1). Impulsive action is the inability to inhibit undesired actions. One of the most frequently used and well-characterized tasks for rats is the five-choice serial reaction time task (5-CSRTT) [34–36] (Fig. 1). In the 5-CSRTT, to obtain a food pellet, the rat is required to perform a nose-poke response in one of five apertures in which a stimulus light located behind the aperture is briefly illuminated (Fig. 1a). The correct response yields a reward at a food magazine. A trial is initiated by the entry of the rat to the food magazine. Following the beginning of a trial and prior to the activation of a stimulus light, there is a 5-s inter-trial interval, during which the rat must refrain from responding at the five-aperture array. Any nose-poke responses to one of the five apertures before the presentation of the stimulus light are characterized as premature responses (Fig. 1b). Premature responses are used as an index of impulsive action. Incorrect responses (i.e., responses to the wrong location after the stimulus is turned on) and omissions [i.e., failure to respond within the limited hold (LH) period] do not indicate impulsive action (Fig. 1b). In the 5-CSRTT, the rat performs to obtain a food reward, and the light stimulus is presented in one of the five apertures as a conditioned reinforcer.

**Table 1:** Definitions and relationships of the terms

Impulsive action	Failure to suppress inappropriate action. Impulsive action occurs due to lack of action inhibition. Action inhibition is classified into “action restraint” and “action cancelation”
Impulsive choice	Tendency to choice of small immediate rewards over larger delayed rewards
Action restraint	Action restraint describes the inhibition of the motor response before that response has been initiated. Action restraint encompasses “waiting to obtain reward” and “waiting to avoid punishment” as defined in this review
Action cancelation	Action cancelation describes the inhibition of a motor response during its execution
Waiting to obtain reward	Suppression of behavior to obtain future reward
Waiting to avoid punishment	Suppression of behavior to avoid future punishment

**Fig. 2:**
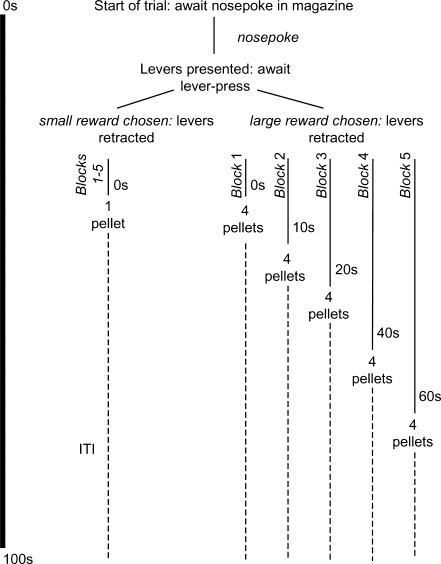
Behavioral procedure of the delay discounting task. The format of a single trial is shown; the trials occurred at 100-s intervals. A session lasted 100 min and consisted of five blocks, each comprising two trials in which only one lever was presented (one trial for each lever in a random order) followed by 10 choice trials. The delay to the large reinforcer was varied systematically across the session. The delays for each block were 0, 10, 20, 40, or 60 s. Modified from [75]

Impulsive choice is the tendency to choose an immediate reward over a delayed reward, even if the delayed reward is known to be larger. One of the most widely used measures of impulsive choice in laboratory animals is the delay-discounting task [12] (Fig. 2). In this task, rats choose between pressing one lever that always results in the immediate delivery of a single food pellet and pressing another lever that always results in four pellets, but only after a delay that is increased progressively across blocks of trials in each session. Impulsive choice, in this paradigm, is commonly attributed to subjects who show a greater choice of the lever giving the smaller, more immediate reward at some or all of the increased delays on the lever giving the larger reward.

The role of the 5-HT system in impulsivity has been studied primarily using forebrain 5-HT depletion and the pharmacological treatment of 5-HT receptors and transporters, yielding contradictory results, particularly in impulsive choice (Table 2). For example, the administration of serotonin selective reuptake inhibitors (SSRIs), which increase extracellular serotonin concentrations, increased the selection rate of a large, delayed reward over a small, immediate reward, meaning a decrease in impulsive choice [9, 10]; in contrast, a nonselective 5-HT antagonist promoted self-controlled choice [12]. Forebrain 5-HT depletion leads to the choice of small, immediate rewards more frequently than large, delayed rewards [9, 11, 18, 27], and systemic treatment with the 5-HT_1A_ agonist 8-hydroxy-2-(di-*n*-propylamino)-tetralin (8-OH-DPAT), which suppresses 5-HT neuronal firing through 5-HT_1A_ autoreceptors, produces impulsive choice [17, 26]. A recent microdialysis study reported a significant increase in 5-HT efflux in the mPFC of rats that were performing the delay-discounting task compared with “yoked” rats, which exercised no choice [24]. However, a recent rodent study demonstrated that the depletion of forebrain 5-HT via the intraventricular administration of the selective neurotoxin 5,7 dihydroxytryptamine (5,7-DHT) produced significant increases in premature responses in the 5-CSRTT but had no effect on the delay-discounting task [14, 15, 22, 23]. An increase in 5-HT release in the mPFC has been correlated with impulsive actions in a visual attention task [37]. Finally, 5-HT_2A_ and 5-HT_2C_ receptor subtypes have been reported to have opposing effects on impulsive choice [13, 16, 19–21, 25]. A potential explanation for these contrasting data is the existence of multiple pre- and post-synaptic 5-HT receptor subtypes in the target areas of 5-HT projections [38] and dynamic compensation mechanisms that are dependent on how 5-HT was manipulated (e.g., by depletion and/or by pharmacological treatment).

**Table 2:** Summary of the role of 5-HT in impulsive action and impulsive choice

Task	Manipulation	Effect	Reference
5-CSRTT	Systemic administration of M100907, 5-HT_2A_ receptor antagonist	Decrease premature responses	[13]
	Systemic administration of DOI, 5-HT_2A_ receptor agonist	Increase premature responses	
	Systemic administration of SB242084, 5-HT_2C_ receptor antagonist	Increase premature responses	
	Systemic administration of Ro60-0175, 5-HT_2C_ receptor agonist	Decrease premature responses	
5-CSRTT	Intra-DRN 5-HT depletion (5,7 dihydroxytryptamine—5,7-DHT)	Increase premature responses	[14]
5-CSRTT	Global 5-HT depletion (5,7-DHT)	Increase premature responses	[15]
5-CSRTT	Intra-mPFC infusion of M100907, 5-HT_2A_ receptor antagonist	Decrease premature responses induced by NMDA receptor antagonist	[16]
5-CSRTT	Systemic and intra-mPFC administration of ketanserin, 5-HT_2A/C_ receptor antagonist	Both systemic and intra-mPFC administration of ketanserin decrease premature responses	[19]
5-CSRTT	Systemic administration of ketanserin, 5-HT_2A/C_ receptor antagonist	Decrease premature responses	[20]
Delay discounting	Systemic administration of ketanserin	No effect	
5-CSRTT	Systemic and intra-mPFC administration of M100907, 5-HT_2A_ antagonist	Decrease premature responses in systemic administration	[21]
		Decrease premature responses in intra-mPFC administration when the stimulus duration was reduced	
5-CSRTT	Systemic administration of M100907, 5-HT_2A_ receptor antagonist	Decreased premature responses	[25]
	Systemic administration of SB242084, 5-HT_2C_ receptor antagonist	Increase premature responses	
T-maze	DRN 5-HT depletion (5,7-DHT)	Prefer no-delay small reward over 15 s delayed large reward (transient)	[9]
	Acute systemic administration of fluoxetin and fluvoxamin (SSRI)	Prefer 25 s delayed large reward over no-delay small reward	
T-maze	Acute systemic administration of citalopram (SSRI)	Prefer 25 s delayed large reward over no-delay small reward	[10]
T-maze	Global 5-HT synthesis inhibition (p-chlorophenylalanine—pCPA)	Prefer no-delay small reward over 15 s delayed large reward	[11]
Delay discounting	Acute systemic administration of citalopram (SSRI)	No effect	[12]
	Acute systemic administration of metergolin, nonselective 5-HT antagonist	Increase self-controlled choice	
Delay discounting	Acute and chronic systemic administration of buspirone, partial 5-HT_1A_ agonist	Increase impulsive choice in acute administration	[17]
		Increase self-controlled choice in chronic administration	
Delay discounting	DRN and MRN 5-HT depletion (5,7-DHT)	Increase impulsive choice	[18]
Probabilistic discounting		No effect	
Delay discounting	Global 5-HT depletion (5,7-DHT)	No effect	[22]
Delay discounting	Global 5-HT depletion (5,7-DHT)	No effect	[23]
Delay discounting	Measurement of 5-HT efflux by microdialysis	Increase mPFC 5-HT efflux during the delay discounting task	[24]
Delay discounting	Systemic administration of 8-OH-DPAT, 5-HT_1A_ receptor agonist	Increase impulsive choice	[26]
	Global 5-HT depletion (5,7-DHT) and systemic administration of 8-OH-DPAT	No effect on 8-OH-DPAT's ability to increase impulsive choice	
Delay discounting	DRN and MRN 5-HT depletion (5,7-DHT)	Increase impulsive choice	[27]

In the 5-CSRTT, to decrease impulsive action, the rats have to wait before making action. In the delay-discounting task, before the rats have to be patient to wait for delayed reward, decision making process according to evaluation of relative value of immediate small reward and delayed large reward is needed. Previous studies have shown that 5-HT manipulations are more effective on impulsive action than on impulsive choice. These results suggest that 5-HT depletion may influence waiting behavior more effectively than decision making process.

## Activation of Serotonin Neurons for Delayed Reward

Previous recording studies of the DRN revealed that the activation of putative 5-HT neurons was correlated with the level of behavioral arousal [39], salient sensory stimuli [40–42], and rhythmic motor outputs [43]. In a recent study, DRN neurons were recorded in monkeys that were performing a reward-oriented saccade task; these neurons exhibited a tonic reward-related response for both small and large rewards [44]. Some neurons showed increased activity during the prediction and receipt of the large reward, whereas other neurons showed increased activity during the prediction and receipt of the small reward. Further analyses revealed that neurons that are tonically excited or inhibited during the task predominantly carried positive reward signals and negative reward signals, respectively [45]. In an odor-guided choice discrimination task, DRN neurons recorded from rats showed that the firing pattern of DRN neurons was correlated with diverse behavioral events, including rewards and conditioned cues [41]. However, no studies have shown a functional link between 5-HT neural activity in the DRN and impulsive behaviors. We sought to provide direct evidence of the involvement of forebrain 5-HT activity in the regulation of impulsive behaviors. Thus, we recorded the firing of 5-HT neurons in the DRN while rats performed a task that required waiting for rewards and a conditioned reinforcer tone [29].

We proposed previously that 5-HT controls the time scale of reward prediction, with increased 5-HT activity promoting the consideration of further delayed rewards in action choice [46]. Furthermore, in a human functional magnetic resonance imaging study, we demonstrated that the DRN was activated when subjects learned to obtain large future rewards [47]. The manipulation of central 5-HT levels via dietary tryptophan depletion and loading has shown that low serotonin levels steepen delayed reward discounting in humans [48]. These results support our serotonin hypothesis. However, there is little direct evidence that 5-HT neural firing and efflux are enhanced by the expectation of a delayed reward as opposed to an immediate reward.

In our pursuit of direct evidence that the DRN 5-HT neurons are activated when animals work for delayed rewards, we previously used in vivo microdialysis to compare 5-HT levels in the DRN of rats that were working for immediate or delayed rewards. Dialysates were collected while the rats performed a task that required waiting for a delayed reward [28]. Serotonin efflux in the rat DRN increased when animals were required to continue poking their nose at the reward site for 4 s and then wait for a delayed reward compared with receiving a reward immediately following a nose poke [28]. Although this result shows that DRN 5-HT neurons are specifically activated in relation to the waiting period for delayed rewards, it remains unclear which behavioral events trigger serotonin neurons to fire, as the temporal resolution of microdialysis measurements is of the order of minutes.

**Fig. 3:**
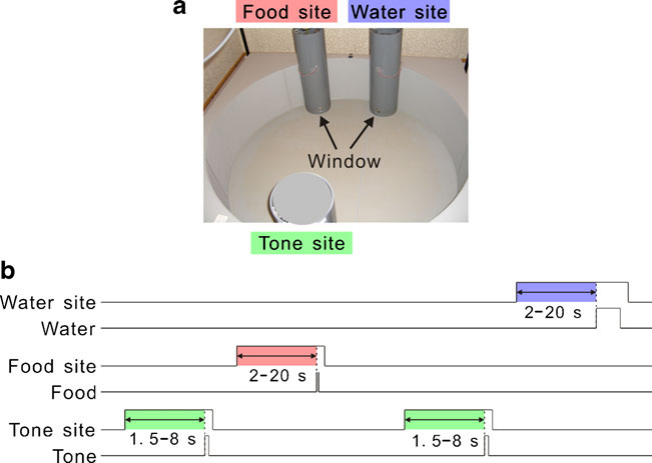
Design of the behavioral task and the rats’ performance. **a** Open-field reward cylinders (food site and water site) and a tone cylinder (tone site) for the task. The windows for nose-pokes (reward locations) are indicated. The tone cylinder also has a small window at the same position as in the reward cylinders. **b** Schematic of the movements required by the rats to receive rewards at the food and water sites. To start the task, the rats have to visit, insert, and keep their noses in the tone site until the tone (8 kHz, 0.4 s) is presented (tone delay). *Green*, *red*, and *blue* areas indicate the tone, food reward, and water reward delays, respectively. See the text for details regarding the tone and reward delay periods. Modified from [29]

To examine how serotonergic neurons respond in real time, we recorded putative serotonergic neurons in the DRN while the rat performed a free operant task that we designated a sequential food–water navigation task [29]. In this task, rats were individually trained and tested in a cylindrical apparatus 1.5 m in diameter with a 45-cm-high wall; three identical-looking cylinders that served as the tone, food, and water sites were fixed in an isosceles triangle (Fig. 3a). This task required the rats to make alternating visits and nose-pokes to the food and water sites via the tone site visit and nose-poke. The rats initiated a trial by maintaining nose-poking in a fixed posture to achieve a continuous interruption of the photo-beam at the tone site during a delay period until a tone (8 kHz, 0.4 s) was presented, thus signaling that a reward was available at one of the reward sites. After the presentation of the tone, the rat was required to continue to nose-poke at one of the reward sites during another delay period until the reward was delivered (Fig. 3b). To continue the task, the rats had to alternately visit two reward sites via the tone site. In the sequential food–water navigation task, the tone worked as a conditioned reinforcer that predicted future food or water rewards. We called the delay periods that preceded the tone and the rewards (food and water) the tone delay and reward delay, respectively.

**Fig. 4:**
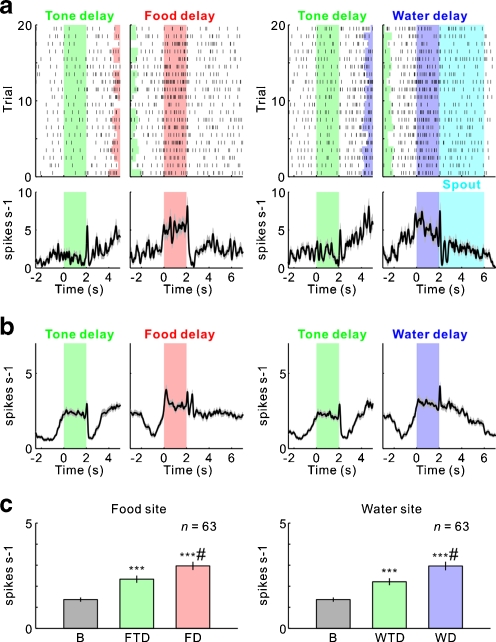
Activity of serotonergic neurons during the tone delay and reward delay periods. **a** Activity of an example neuron recorded in the dorsal raphe nucleus is shown separately for food (*left*) and water (*right*) during the sequential food–water navigation task in which the waiting periods for tone (tone delay) and for rewards (food and water delay) are 2 s (the constant delay condition). For each reward, raster plots of neural activity (*top*) and peri-event time histograms smoothed with a Gaussian filter (SD = 50 ms) (*bottom*) are aligned at the time of the tone site entry (*left*) and at the time of the reward site entry (*right*). The raster plots represent neural activity in the order of the occurrence of trials for each reward site from *bottom* to *top*. Each *dot* represents a spike. The tones for the food and water sites are the food tone and water tone, respectively. *Green*, *red*, and *blue* areas indicate the tone delay, food delay, and water delay periods, respectively. *Light blue* areas indicate the water spout presenting period. **b** Average activity of the 63 neurons recorded during the constant delay condition. **c** Average firing rate during the tone and reward delay periods. Average firing rates during the baseline (*B*), food tone delay (*FTD*), water tone delay (*WTD*), food delay (*FD*), and water delay (*WD*) periods are shown. *Asterisks* (***) indicate significant differences relative to baseline activity (Wilcoxon signed-rank test, *p* < 0.0001). *Hash marks* (^#^) indicate significant differences relative to tone delay activity (Wilcoxon signed-rank test, *p* < 0.0001). In (**a**) and (**b**), the *gray shading* indicates SEM. Modified from [29]

**Fig. 5:**
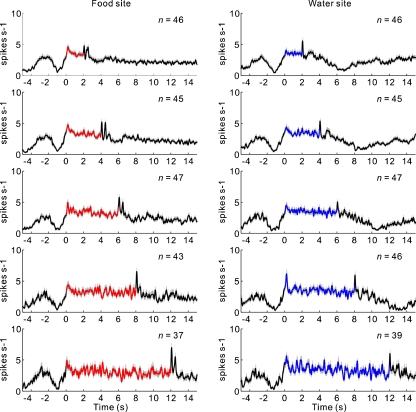
Population activity of DRN serotonin neurons under an extended reward delay condition in which the tone delay was fixed at 1.5 s and the reward delay at both sites was increased gradually every 300 s (2, 4, 6, 8, 12 s). Averaged activity of 5-HT neurons aligned to the time of entry to the food site (*left*) and water site (*right*). *Red* and *blue* lines indicate activity during the food and water delay periods, respectively. *Gray shadings* represent SEM. Food site: 2-s delay (*n* = 46), 4-s delay (*n* = 45), 6-s delay (*n* = 47), 8-s delay (*n* = 43), and 12-s delay (*n* = 37). Water site: 2-s delay (*n* = 46), 4-s delay (*n* = 45), 6-s delay (*n* = 47), 8-s delay (*n* = 46), and 12-s delay (*n* = 39). Modified from [29]

**Fig. 6:**
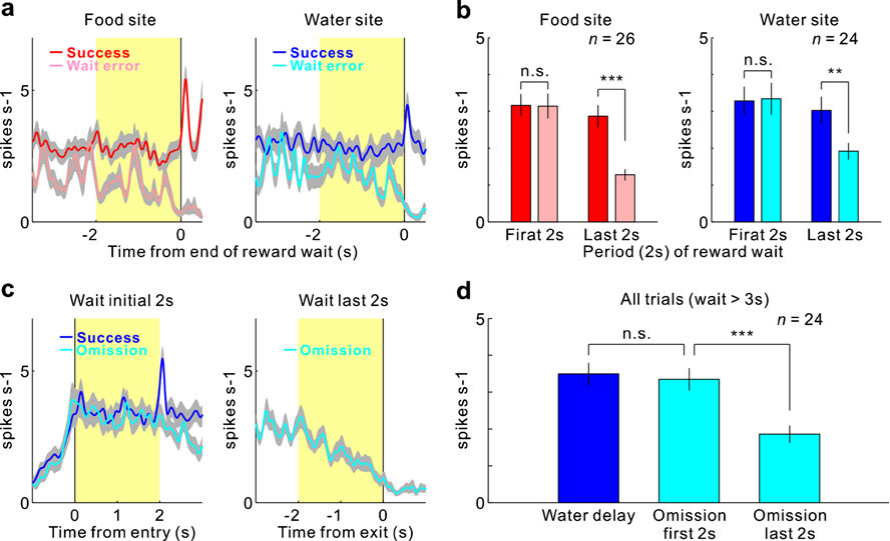
Activity of 5-HT neurons during reward wait error (i.e., failure to wait for delayed rewards) in the extended reward delay test and during water reward omission. **a** Population activity aligned to the onset of the reward presentation (*red*, food; *blue*, water) and to the reward wait error (*pink*, food wait error; *cyan*, water wait error) (*left*, food site, *n* = 26; *right*, water site, *n* = 24). *Gray shadings* represent SEM. *Light yellow areas* indicate the periods that were used to analyze the average firing rate. **b** Average firing rate during the first and last 2 s of the waiting period after entry into the reward site in the case of a successful entry (*red*, food; *blue*, water) or in the case of a wait error entry (*pink*, food wait error; *cyan*, water wait error) (*left*, food site, *n* = 26; *right*, water site, *n* = 24; ±SEM). **c** Population activity aligned to water site rewarded entry (*blue*) and to water omission entry (*cyan*) (*left*) (*n* = 24). Population activity aligned to water site exit after water omission entry (*right*) (*n* = 24). *Gray shadings* represent SEM. *Light yellow areas* indicate the periods that were used to analyze the average firing rate. **d** Average firing rates during a 2-s period following water site rewarded entry, after water omission entry, and before water site exit (*n* = 24; ±SEM). *p < 0.01, **p < 0.001, ***p < 0.0001; Wilcoxon signed-rank test. *n.s.* not significant. Modified from [29]

We found that many 5-HT neurons exhibited an increase in tonic activity during the period in which the rat waited for forthcoming rewards [29] (Fig. 4a, b). These results revealed that the waiting behavior for delayed rewards was the crucial behavioral event for activating 5-HT neurons in the DRN. To investigate further how 5-HT neural activity is related to waiting behavior for delayed rewards, we compared the neural activity of rats that were waiting for delayed rewards with a conditioned reinforcer tone [29] (Fig. 4c). The sustained 5-HT neural activity during the reward delay period was significantly higher than the activity during the tone delay period, which suggests that this increased activity was not attributable simply to the nose-poking behavior, which was required for both the reward and tone sites. When the reward and tone delays were independently extended (an extended reward or tone delay test), tonic firing persisted until the delivery of the reward or tone, and the rats waited longer for primary rewards than for the conditioned reinforcer tone [29] (Fig. 5). When the reward delay was gradually prolonged during the extended reward delay test, the number of failures to wait for delayed rewards (rewards wait error) gradually increased, and 5-HT neural activity ceased before the rats ceased waiting for possible future rewards [29] (Fig. 6a, b). When an expected water reward was suddenly omitted for several continuous trials (i.e., a water omission test), 5-HT neural activity also dropped preceding the exit from the water site during adaptively truncated waiting in the water omission trials [29] (Fig. 6c, d). These results suggest that an increase in 5-HT neuronal firing facilitates a rat’s waiting behavior with the prospect of forthcoming rewards and that higher serotonin activation enables longer waiting periods.

## Waiting to Obtain Reward and Waiting to Avoid Punishment

Classic theories suggest that central 5-HT neurons are involved in the behavioral inhibition that is associated with the prediction of negative rewards or punishment [30–33]. In both pharmacological treatment and lesion studies that decrease 5-HT transmission, animals exhibited a deficit in passive avoidance in which animals learned to suppress their natural tendency to enter a dark chamber from a light chamber after they experienced aversive stimuli such as a foot shock in the dark chamber [33]. Dietary tryptophan depletion abolished the punishment-induced slowing of reaction times for the go responses in a go/no-go task in healthy volunteers. The go responses in control subjects became slower when incorrect go responses evoked a large punishment compared with conditions in which correct go responses earned a large reward. This punishment-induced inhibition of responding was absent following tryptophan depletion [49].

We found that 5-HT neural activity increased when rats waited for delayed rewards. These results suggest that the 5-HT system contributes to the modulation of patience for the attainment of rewards. In this article, we propose that the 5-HT system is involved in the decrease of behavioral activity both to avoid aversive events with a prediction of punishment as well as to achieve rewards with a prediction of reward. To clarify the decrease in these two behavioral activities, we defined the behavior as either “waiting to obtain reward” when they decreased their activity to obtain a reward or “waiting to avoid punishment” when the animals suppressed their activity to prevent future punishment.

In our task, maintaining nose-poking for a delayed reward is the “waiting to obtain reward”. When the expected water reward was suddenly omitted for several consecutive trials, the duration of nose-poking gradually shortened [29]. This result suggests that rats maintained their nose-poking behavior at reward sites to receive rewards when they predict that a reward is forthcoming.

In the 5-CSRTT, the requirement that the rat withhold nose-poke responses in one of the five apertures until an internal stimulus light is briefly illuminated is the “waiting to obtain reward”. The increase in premature responses after forebrain 5-HT depletion might result from an inability to wait for the visual targets that act as a conditioned reinforcer and not from fear or the prediction of a 5-s time-out following a premature response, because if the rats are patient and wait for the visual targets, they receive the reward. If the intent of the rats is to avoid time-out and not wait for the visual targets, the rats might remain motionless after the presentation of the stimulus light. This waiting for visual targets in the 5-CSRTT resembles the waiting for tones that is observed in the sequential food–water navigation task in which 5-HT neural activity increases owing to both of the stimuli working as conditioned reinforcers. In the 5-CSRTT, 5-HT neurons might increase their firing rate while the rat is waiting for the visual target that is presented in one of the five apertures.

Similar to behavioral inhibition, the term “action inhibition” is used to explain the inhibitory control of animal behavior. Impulsive action occurs due to lack of action inhibition. Action inhibition can be divided into action restraint and action cancellation [50] (Table 1). Action restraint describes the inhibition of the motor response before the response has been initiated. Action restraint is studied using tasks such as the go/no-go task, and the main focus is the ability or failure to withhold responding [51]. Action cancellation indicates the inhibition of a motor response that was already initiated during the execution of the motor response. Action cancellation is studied using the stop-signal reaction time task (SSRTT) in which the stop signal is presented to inhibit the ongoing go response following the presentation of go signal [51]. Action restraint encompasses both “waiting to obtain reward” and “waiting to avoid punishment” as defined in this article (Table 1).

In the SSRTT, the manipulation of 5-HT levels in either rats or humans does not affect performance when the previously initiated go response is required to stop by the tone signal [51–55]. However, 5-HT depletion impairs task performance when the tone signal is presented without a delay at the start of the go response and when the time period during which the rat is required to withhold the go response is extended [55]. The inhibition of the response in the SSRTT that is induced by the simultaneous presentation of the go signal and stop signal resembles the no-go trial of the go/no-go task. In rats, 5-HT depletion impairs waiting but not the stop-signal reaction time, which supports a role of 5-HT in the “waiting to obtain reward”, as in the SSRTT, the success to withhold response is rewarded [55].

Serotonin has also been implicated in inhibitory control in the go/no-go task [56, 57]. When a correct no-go response is rewarded, withholding the go response would be the “waiting to obtain reward”, as the animals execute a no-go trial while predicting a future reward. In contrast, if an incorrect no-go response is punished, withholding the go response would be the “waiting to avoid punishment”, as the animals inhibit a go response to avoid punishment. In a symmetrically reinforced go/no-go conditional visual discrimination task, global 5-HT depletion using 5,7-DHT fails to acquire visual discrimination due to an inability to withhold responding to a no-go signal and also impairs the ability of previously trained rats to subsequently inhibit correctly to the no-go signal [56]. This inability to withhold the response in no-go trials can be explained by an impairment of the “waiting to obtain reward”. Rats that receive para-chloroamphetamine to induce 5-HT depletion within the brain show impaired acquisition of a go/no-go visual discrimination task in which the go responses during the light and dark phases are rewarded and non-rewarded, respectively [57]. In this study, withholding the go response during the dark phase is the “waiting to avoid punishment”.

Depleting 5-HT by a median (but not dorsal) raphe injection of 5,7-DHT impairs the acquisition and performance of behaviors that are maintained under a differential reinforcement of a low-rate (DRL) schedule of reinforcement [58]. During the DRL schedule of reinforcement, operant responses are reinforced only when they occur after a fixed interval (e.g., 20 s, as in a DRL 20-s schedule) following the previous rewarded response. Similarity between premature responses in the 5-CSRTT and non-rewarded operant responses in the DRL schedule has been suggested, as waiting for a defined temporal interval is required for reinforcement in both tasks [6]. A primary difference between the two tasks is that the 5-CSRTT, uses explicit signals—the stimulus light at one of the five apertures—that predict future rewards. On the other hand, the DRL schedule has no explicit signal for future rewards. The lack of an effect on behavior following dorsal raphe 5,7-DHT lesions may be due to a lack of explicit goal expectations (rewards or conditioned reinforcers) that can be obtained after waiting, as waiting is not in itself sufficient to obtain rewards or conditioned reinforcers in the DRL schedule.

How is 5-HT neural activity related to the “waiting to obtain reward” and “waiting to avoid punishment”? Does the same DRN 5-HT neuron contribute to both the “waiting to obtain reward” and “waiting to avoid punishment”? Alternatively, do 5-HT neurons differently regulate the “waiting to obtain reward” and “waiting to avoid punishment”? Although no study has examined how 5-HT neurons respond during the “waiting to avoid punishment”, 5-HT neurons might increase their firing rate during the “waiting to avoid punishment”. If the same rat can learn that the same behavior (such as maintaining nose-poking causes reward gain or punishment avoidance, depending on the situation), we could examine how the activity of a single 5-HT neuron responds while “waiting to obtain reward” and “waiting to avoid punishment”.

We would like to propose a task for this purpose. In a tone discrimination task, tone 1 and tone 2 are associated with a reward and punishment (e.g., an electric shock), respectively. After the presentation of tone 1, the rat can receive a reward by maintaining nose-poking in a reward site for several seconds. This nose-poke behavior is the “waiting to obtain reward”. The rat can avoid punishment by maintaining nose-poking in a safe site for several seconds after the presentation of tone 2. In this case, the nose-poke behavior is the “waiting to avoid punishment”. During the task, the rat would wait for the reward in the positive reward prediction and inhibit its behavior to avoid aversive stimuli with a negative reward expectation. Unit recordings of 5-HT neurons from this rat would reveal whether the same 5-HT neurons are related to the “waiting to obtain reward” and the “waiting to avoid punishment” or whether the “waiting to obtain reward” and the “waiting to avoid punishment” are regulated separately by different 5-HT neurons. Furthermore, to examine which neural circuits regulate the “waiting to obtain reward” and “waiting to avoid punishment”, it is important to examine the projections of the 5-HT neurons that respond during the “waiting to obtain reward” and/or the “waiting to avoid punishment”. Electrical stimulation of these projection sites to produce antidromic activation may reveal the areas that are influenced by 5-HT.

## Putative Role of Serotonin for the Regulation of Patience for Future Rewards

The neural circuitry that mediates the “waiting to obtain reward” might be related to patience with respect to future rewards. What are the neural substrates of the “waiting to obtain reward”, and how does 5-HT influence these neural circuits? First, the NAcc contributes to the “waiting to obtain reward”. Evidence from lesion studies suggests that the core region of the NAcc contributes to both DRL response inhibition and to premature responses in the 5-CSRTT [59, 60]. Recent studies have shown that systemic application of 5-HT_2A_ receptor antagonists causes a reduction in impulsive action, whereas 5-HT_2C_ receptor antagonists cause an increase in impulsivity in the 5-CSRTT [13, 16, 19, 21]. Intra-NAcc infusion of the 5-HT_2A_ receptor antagonist M100907 and the 5-HT_2C_ receptor antagonist SB242084 significantly decrease and increase, respectively, the premature responses in the 5-CSRTT [61]. The integrity of the NAcc is necessary for the prevention of premature responses during the anticipation or waiting for reward presentation periods. Unit recording studies have revealed that neurons in the NAcc exhibit an anticipatory response to delayed rewards during waiting [62, 63].

Second, the mPFC and OFC also contribute to the waiting to obtain reward behavior. Excitotoxic lesions of the infralimbic PFC, the ventral part of the mPFC, and the OFC induce premature responses in the 5-CSRTT [64]. Intra-mPFC infusion of the 5-HT_2A_ antagonist M100907 decreases premature responses in the 5-CSRTT when the duration of the visual target is shortened [21]. However, no effect of either M100907 or the 5-HT_2C_ receptor antagonist SB242084 on premature responses with standard task parameters in the 5-CSRTT are observed with intra-mPFC infusions [61]. Blocking NMDA receptors in the mPFC by intracortical infusion of 3-(*R*)-2-carboxypiperazin-4-propyl-1-phosphonic acid (CPP) markedly and reliably enhance extracellular glutamate [65, 66] and increase premature responses in the 5-CSRTT [67]. The increase in premature responses that is induced by intra-mPFC CPP infusion is prevented by the systemic administration of the 5-HT_2C_ receptor agonist Ro60-0175 [68]. A recent study showed that 5-HT_2C_ receptors are located in GABAergic interneurons within the mPFC, in particular, in neurons containing the calcium-binding protein palvalbumin [69]. This result suggests that an increase in GABAergic tone mediated by 5-HT_2C_ receptors in the mPFC contributes to the suppression of CPP-induced glutamate release and an increase in premature responses.

In the OFC and mPFC, a sustained increase in activity has been observed during waiting for delayed rewards [70–73]. These neural activities may interact with the activity of 5-HT neurons in the DRN. The role of the OFC is to signal expected outcomes to projection regions but not to contribute directly to response inhibition [73]. Recently, the firing rates of many single neurons in the OFC were shown to represent the confidence of decision making when decision difficulty was manipulated by varying the distance between the stimuli and the category boundary [74]. When tested in a delayed reward version of the task, the willingness of the rats to wait for rewards increased with confidence [74]. An explicit representation of the goal and/or the value of the goal would be important in the learning of patience to receive future rewards. Confidence and/or reward expectation would be helpful in patience for delayed rewards, as explicit representations of goals would enable animals to be patient while waiting for future rewards. These confidence-related signals may influence 5-HT neural activity during the “waiting to obtain reward”.

It remains unclear how confidence and/or the explicit representation of goals modulate 5-HT neural activity and how 5-HT neural activity influences the neural activity of projection sites. Simultaneous recordings from the OFC/mPFC, NAcc, and DRN would help to examine the contribution of these regions to the “waiting to obtain reward”. For example, how is the neural activity of these regions correlated with an animal’s behavior when it stops waiting for the delayed reward? Moreover, how does neural activity change according to changes in the animal’s behavior during manipulations of confidence and the “waiting to obtain reward”?

## Conclusion

**Fig. 7:**
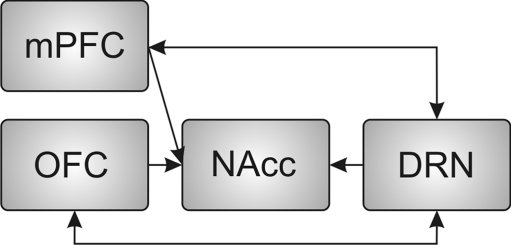
Putative neural circuit that contributes to the “waiting to obtain reward”. DRN 5-HT neurons project to the NAcc, mPFC, and OFC. Previous studies revealed that 5-HT in the NAcc and mPFC contributes to regulation of impulsive action. The OFC may transmit confidence and/or reward expectation signals to influence 5-HT neural activity during the “waiting to obtain reward”

It is well established that the 5-HT system contributes to “waiting to avoid punishment” when there is the prospect of future punishment. In this article, we propose that the 5-HT system also plays a role in “waiting to obtain reward”, which is a waiting behavior with the purpose of receiving future rewards. Interactions among the OFC, mPFC, NAcc, and 5-HT neurons in the DRN could be involved in the waiting to obtain reward behavior (Fig. 7). Neural circuits for the “waiting to obtain reward” might regulate patience while waiting for future rewards. Clarifying the neural mechanism in the “waiting to obtain reward” would be beneficial for the clinical treatment of patients who lack the patience to wait for delayed rewards: for example, individuals with attention deficit/hyperactivity disorder or drug addiction. Further study is needed to determine how 5-HT efferents modulate cellular and network properties to facilitate the “waiting to obtain reward” and how the afferents to the DRN regulate 5-HT neural activities.
